# Plugging in or going wireless: strategies for interspecies electron transfer

**DOI:** 10.3389/fmicb.2014.00237

**Published:** 2014-05-16

**Authors:** Pravin Malla Shrestha, Amelia-Elena Rotaru

**Affiliations:** ^1^Department of Microbiology, University of MassachusettsAmherst, MA, USA; ^2^Energy Biosciences Institute, University of CaliforniaBerkeley, CA, USA; ^3^Nordic Center for Earth Evolution, University of Southern DenmarkOdense, Denmark

**Keywords:** syntrophy, diet, interspecies electron transfer, conductive pili, coculture

## Abstract

Interspecies exchange of electrons enables a diversity of microbial communities to gain energy from reactions that no one microbe can catalyze. The first recognized strategies for interspecies electron transfer were those that relied on chemical intermediates that are recycled through oxidized and reduced forms. Well-studied examples are interspecies H_2_ transfer and the cycling of sulfur intermediates in anaerobic photosynthetic communities. Direct interspecies electron transfer (DIET) in which two species establish electrical contact is an alternative. Electrical contacts documented to date include electrically conductive pili, as well as conductive iron minerals and conductive carbon moieties such as activated carbon and biochar. Interspecies electron transfer is central to the functioning of methane-producing microbial communities. The importance of interspecies H_2_ transfer in many methanogenic communities is clear, but under some circumstances DIET predominates. It is expected that further mechanistic studies and broadening investigations to a wider range of environments will help elucidate the factors that favor specific forms of interspecies electron exchange under different environmental conditions.

## INTRODUCTION

**FIGURE 1:**
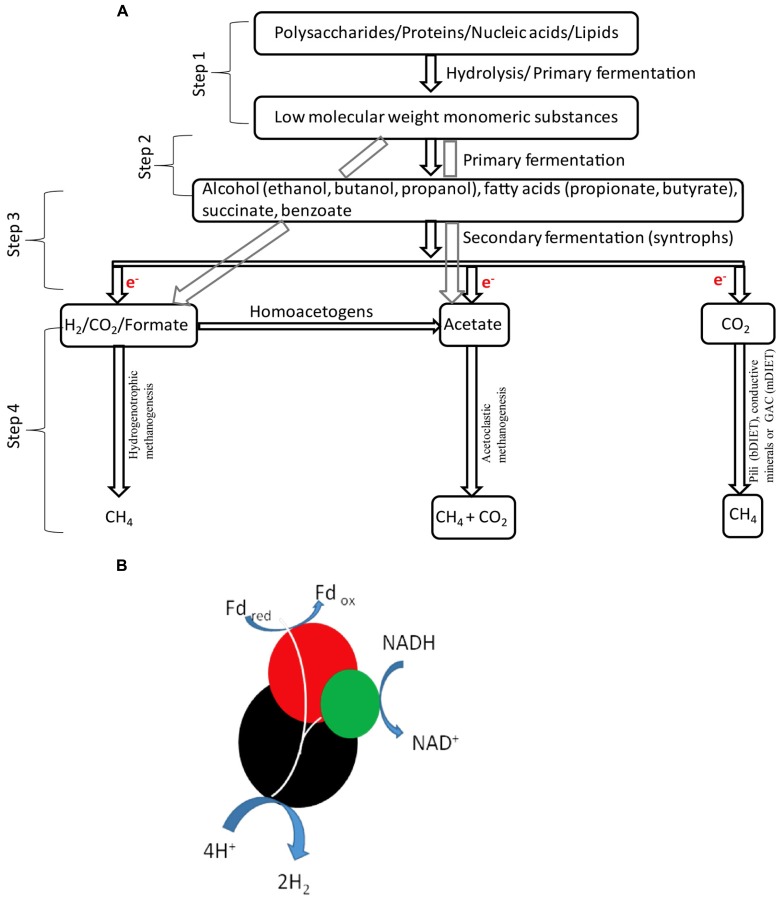
** Organic matter degradation in methanogenic environments (A)**. Sketch of the coupling of H_2_ with the energetically favorable oxidation of a reduced ferredoxin in the presence of putative NADH-linked confurcating hydrogenases [modified from McInerney et al., 2011; **(B)**].

Interspecies electron transfer plays a key role in the functioning of methane-producing microbial communities, which have a significant impact on the global carbon cycle (Stams and Plugge, 2009; Sieber et al., 2012). Organic matter mineralization to methane by microbial processes contributes to 69% of the atmospheric CH_4_ (Conrad, 2009) and it involves four major steps (**Figure 1A**):

(1) Hydrolytic bacteria break down complex compounds such as polysaccharides, proteins, nucleic acids, and lipids to monomeric substances (Schink and Stams, 2013), (2) Primary fermenters convert monomeric substances to H_2_/formate, CO_2_ and small organic molecules such as lactate, succinate, fatty acids, and acetate (Morris et al., 2013; Schink and Stams, 2013), (3) Syntrophic bacteria carryout secondary fermentation of small organic molecules to produce acetate, formate, H_2_ and CO_2_ (Morris et al., 2013; Schink and Stams, 2013), or releases electrons for direct electric connections (Summers et al., 2010; Rotaru et al., 2014), (4) Methanogenic Archaea uses electrons from H_2_/formate/shuttles or directly to reduce CO_2_ to CH_4_ (Morris et al., 2013; Rotaru et al., 2014; Sieber et al., 2014).

Interspecies electron transfer via H_2_/formate has been extensively reviewed in recent years (Morris et al., 2013; Schink and Stams, 2013; Sieber et al., 2014). Besides, H_2_/formate, there are many important mechanisms of interspecies electron transfer reported, which include but are not limited to pili mediated direct interspecies electron transfer (DIET; Summers et al., 2010; Morita et al., 2011; Nagarajan et al., 2013; Shrestha et al., 2013a, b; Rotaru et al., 2014) and mineral mediated direct intrespecies electron transfer (Kato et al., 2012a, b; Liu et al., 2012, 2014; Chen et al., 2014), or by shuttle molecules like cysteine (Kaden et al., 2002), sulfur compounds (Biebl and Pfennig, 1978; Milucka et al., 2012), and humics (Lovley et al., 1999; Liu et al., 2012). This review discusses recent findings on interspecies electron transfer during syntrophic interactions, with the main focus on DIET mechanisms.

## H_2_ AND FORMATE AS ELECTRON TRANSFER MOLECULES

H_2_ and formate are important electron transfer molecules that are reported in various methanogenic environments (Schink and Stams, 2006, 2013; Stams and Plugge, 2009), these are described briefly under separate headings below:

### H_2_ AS ELECTRON TRANSFER MOLECULE

Interspecies electron transfer via H_2_ was first demonstrated almost four decades ago in a defined co-culture (Bryant et al., 1967) of the “S organism,” which converted ethanol to acetate and H_2_, only in the presence of *Methanobacterium ruminantium*, which consumed H_2_ for the reduction of CO_2_ to CH_4_ (Bryant et al., 1967). H_2_ is a very powerful electron donor under anoxic conditions and must be continuously removed by partner organism in order for the syntrophic interaction to take place (Nedwell and Banat, 1981; Lovley and Ferry, 1985; Kleerebezem et al., 1999; Wintermute and Silver, 2010). The generation of H_2_ is energetically unfavorable at H_2_ partial pressures above 10^-^^3^ bar (Schink and Stams, 2013), however, syntrophic microorganisms bypass this energetic barrier by coupling the unfavorable H_2_ production with the energetically favorable oxidation of a reduced compound like ferrodoxin (**Figure 1B**), a process known as electron confurcation (Schut and Adams, 2009; Sieber et al., 2010, 2012). Confurcating hydrogenases are found in the genomes of all H_2_ generating syntrophs described to date (Sieber et al., 2010, 2012).

### FORMATE AS ELECTRON TRANSFER MOLECULE

**FIGURE 2:**
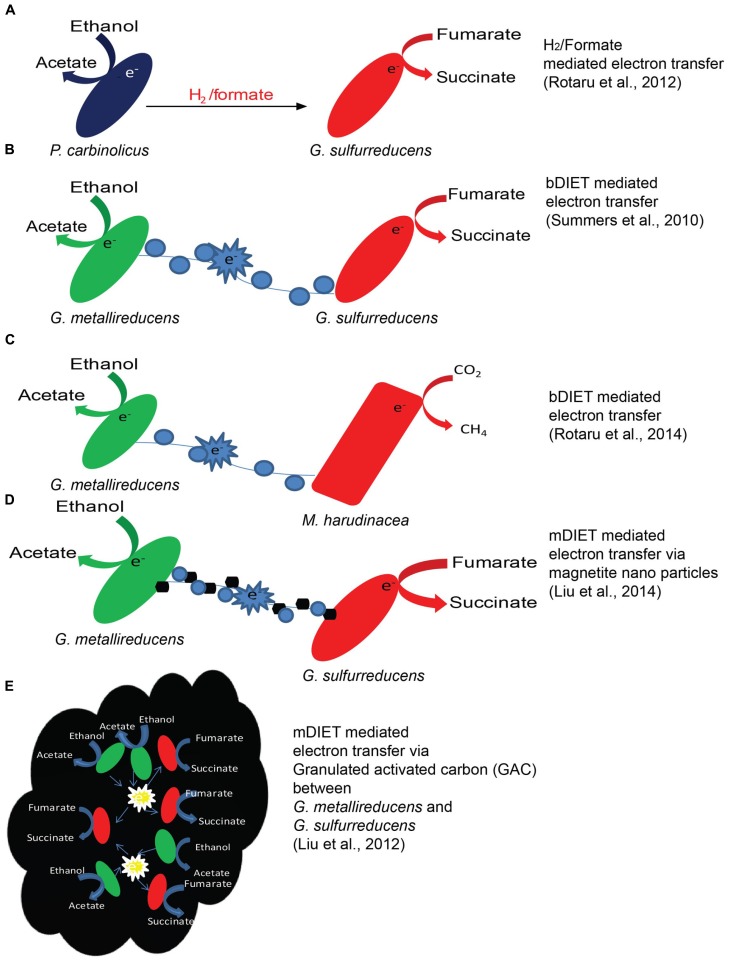
**Examples of mechanisms of electron transfer**. H_2_ transfer between *P. carbinolicus* and *G. sulfurreducens* **(A)**, bDIET between *G. metallireducens* and *G. sulfurreducens* **(B)**, mineral mediated mDIET between *G. metallireducens* and *G. sulfurreducens* with nano-sized minerals **(D)** or GAC **(E)** in the presence of ethanol as the electron donor and fumarate as the electron acceptor. DIET in a co-culture of *G. metallireducens* and *Methanosaeta harudinacea* where ethanol was used as electron donor and CO_2_ is reduced to CH_4_ by *Methanosaeta* using electrons received directly from *G. metallireducens* via bDIET **(C)**.

Formate is an alternative to H_2_ and could also act as an electron carrier between syntrophic partners (Thiele and Zeikus, 1988; Boone et al., 1989; Hattori et al., 2001; de Bok et al., 2004; Stams et al., 2006; Stams and Plugge, 2009). The use of formate as an electron transfer molecule has been noticed especially in co-cultures thriving on proteins (Zindel et al., 1988) or fatty acids like propionate and butyrate (de Bok et al., 2004; Sousa et al., 2007). Certain communities might favor formate transfer because formate has ca. three times higher diffusion coefficient as compared to H_2_, and allows larger mass transfer to methanogens (Boone et al., 1989). It has been also reported that some syntrophic interactions uses both formate and H_2_ to transfer electrons between species (Boone et al., 1989; Dong and Stams, 1995; Stams et al., 2006; Rotaru et al., 2012). This dual mechanism of electron transfer using H_2_ and formate (**Figure 2A**) has been studied in detail using deletion mutants, in a co-culture of *Pelobacter carbinolicus* and *Geobacter sulfurreducens* (Rotaru et al., 2012). For example, when a co-culture was established with a hydrogenase mutant (*hybL*) of *G. sulfurreducens*, the formate dehydrogenase (*fdnG*) gene of *G. sulfurreducens* was over-expressed (Rotaru et al., 2012).

## ELECTRON TRANSFER VIA SHUTTLE MOLECULES

Electron shuttles are chemical compounds that facilitates the transfer of electrons to and from bacteria these may include sulfur compounds (Biebl and Pfennig, 1978), humic substances (Lovley et al., 1996, 1998, 1999; Newman and Kolter, 2000), and flavins (Marsili et al., 2008; von Canstein et al., 2008; Brutinel and Gralnick, 2012), etc.

### SULFUR COMPOUNDS AS MEDIATORS FOR INTERSPECIES ELECTRON TRANSFER

Sulfur compounds as shuttle were first discovered between green sulfur bacteria and sulfate-reducing bacteria (SRB; Biebl and Pfennig, 1978). *S*(0) is converted to sulfide by a sulfate reducing bacteria and then recycled back to *S*(0) by a photosynthetic green-sulfur bacteria creating an interspecies S-cycle (Biebl and Pfennig, 1978). The second discovered S-based interspecies interaction used cysteine as electron shuttle between *G. sulfurreducens* and *Wolinella succinogenes*, growing with acetate as electron donor and nitrate as electron acceptor (Kaden et al., 2002). S-compounds were also found responsible for electron transfer between anaerobic methane oxidizing *Archaea* (ANME) and sulfate reducing bacteria (Boetius et al., 2000), which oxidizes methane with sulfate, one of the most studied, yet least understood interactions. The members of the anaerobic oxidation of methane consortia were initially thought to exchange electrons via methyl-sulfides (Moran et al., 2008), however, more recently the electron carrier within the consortium was revealed to be polysulfides (Milucka et al., 2012).

### HUMICS AND HUMICS EQUIVALENTS AS ELECTRON SHUTTLES

Humic substances are ubiquitous in nature (Lovley et al., 1996; Bittner et al., 2007). The humic substance analog, anthraquinone disulphonate (AQDS) serves as an electron shuttles between *G. metallireducens* and *G. sulfurreducens* (Liu et al., 2012), or between *G. metallireducens* and *W. succinogenes* (Lovley et al., 1999). This came as no surprise because it is known that certain microorganisms can use AH_2_QDS as electron donor (Lovley et al., 1999), while others use AQDS as electron acceptor (Lovley et al., 1996). However, AQDS cannot mediate electron transfer in *G. metallireducens* and *M. barkeri* co-cultures, likely because of the redox potential of the AQDS couple is too high to reduce carbon (E0’ = -184 mV) to reduce carbon dioxide to methane (E0’ = -240 mV; Liu et al., 2012).

### FLAVINS AS ELECTRON SHUTTLES

Flavins were also noted to improve electron transfer to electrodes in *Shewanella* biofilms (Marsili et al., 2008; von Canstein et al., 2008; Brutinel and Gralnick, 2012) yet their impact on interspecies interactions remains to be reported.

## DIRECT INTERSPECIES ELECTRON TRANSFER

To clearly distinguish between conductive mineral mediated DIET and direct cell contact DIET, we have subcategorized the pili mediated electron transfer, as biological DIET (bDIET), and the conductive mineral mediated DIET, as mineral DIET (mDIET).

### BIOLOGICAL DIET

Biological DIET (**Figures 2B,C**) was first described in *G. metallireducens* and *G. sulfurreducens* co-cultures, growing in a defined minimal medium with ethanol as electron donor and fumarate as electron acceptor (Summers et al., 2010). Tightly associated aggregates were consistently noticed in co-cultures growing via bDIET (Summers et al., 2010; Shrestha et al., 2013a; Rotaru et al., 2014) but not during growth via H_2_/formate electron transfer (Rotaru et al., 2012). The mechanism for bDIET in *Geobacter* co-cultures was intensely studied during the past few years, combining phenotypic, genetic, transcriptomics, proteomics analysis (Summers et al., 2010; Shrestha et al., 2013a, b). bDIET might be favored over H_2_ or formate transfer under certain conditions (Lovley, 2011) as demonstrated using genome-scale models including genomic, transcriptomic and physiological data (Nagarajan et al., 2013). The absence of H_2_/formate mediated electron transfer in the co-culture was best shown by the ability of *G. metallireducens* to generate successful syntrophic co-cultures with a double mutant of *G. sulfurreducens* (*ΔhybLΔfdnG*) incapable of H_2_ or formate uptake (Rotaru et al., 2012). Furthermore, bDIET is seemingly capable to produce successful co-cultures in the absence of acetate transfer as supportive mechanism of electron exchange as revealed in a recent study (Shrestha et al., 2013a) in co-cultures of *G. metallireducens* with strain of *G. sulfurreducens* depleted in acetate utilization capacity, a citrate synthase mutant (*ΔgltA*; Ueki and Lovley, 2010). This study clearly revealed that bDIET alone is sufficient for energy conservation in syntrophic co-cultures.

Biological DIET interactions with fumarate as terminal electron acceptors are probably not ecologically relevant, but more recently bDIET was discovered in co-cultures of *G. metallireducens* with *Methanosaeta harudinacea* (Rotaru et al., 2014). These two genera of methanogens are responsible for the majority of methane emission in environments such as paddy soils (Grosskopf et al., 1998; Feng et al., 2013) or anaerobic digesters (Vavilin et al., 2008; Morita et al., 2011; Rotaru et al., 2014; Ying et al., 2014). Only these acetoclastic methanogens were capable of bDIET-interactions with *G. metallireducens*, whereas hydrogenotrophic methanogens were not (Rotaru et al., 2014). *Methanosaeta* was shown to use electrons directly for the reduction of CO_2_ to methane because the methanogen converted 1/3 of the ^1^^4^C-bicarbonate to ^1^^4^C methane (Rotaru et al., 2014). Other shuttles were excluded as electron transferring mechanisms because a pili-deficient *G. metallireducens* could not produce successful co-cultures with *Methanosaeta* or *Methanosarcina* (Rotaru et al., 2014).

#### Role of pili in bDIET

Pili are known to have an important role in biofilm formation (Moreira et al., 2006; Reguera et al., 2007; Oxaran et al., 2012; Snider et al., 2012), but also for the conductive properties of *Geobacter* biofilms (Summers et al., 2010; Malvankar et al., 2011; Malvankar and Lovley, 2012, 2014; Vargas et al., 2013). Co-cultures do not grow when initiated with a strain of either *G. metallireducens* (Summers et al., 2010) or *G. sulfurreducens* (Rotaru et al., 2014) in which the gene for PilA is deleted, confirming the importance of conductive pili (Reguera et al., 2005, 2006; Lovley, 2011; Malvankar et al., 2011) networks for bDIET. It has been proposed that the stacking of π–π orbitals of five aromatic amino-acids in the carboxyl-terminus of PilA, the pilin monomer, contribute to the metallic-like conductivity similar to that of conductive organic polymers (Vargas et al., 2013). A *G. sulfurreducens* strain deficient in the five aromatic amino acids (ARO5), the pili were still produced with properly localized OmcS and yet the biofilms of ARO5 showed greatly diminished conductivity (Vargas et al., 2013). In another study, the gene for conductive pili in *G. sulfurreducens* was replaced with the non-conductive *pilA* gene of *Pseudomonas aeruginosa* PAO1 (Liu et al., 2013) generating a mutant strain PAO1, which can express properly assembled *P. aeruginosa* pili ornamented by outer surface c-type cytochromes. However, PAO1 biofilms had significantly lower conductivity than wild type *G. sulfurreducens* and was unable to reduce Fe^3^^+^-oxides or produce current (Liu et al., 2013). The lack of conductivity in PAO1 biofilms indicates that three out of five aromatic amino acids at the C-terminus domain are necessary for conductivity (Liu et al., 2013). These findings validated that OmcS alone on scaffold-pili is insufficient to confer conductivity to *Geobacter* biofilms, in contrast to a recent hypothesis, which suggested that conductivity is the result of electron-hopping via cytochromes aligned on the pili of *G. sulfurreducens* (Strycharz-Glaven et al., 2011).

#### Role of cytochromes in bDIET

*Geobacter sulfurreducens* was used as model organism for the study of extracellular electron transfer, and several studies revealed that besides pili, *G. sulfurreducens* require a multitude of extracellular and periplasmic cytochromes for insoluble Fe^3^^+^ oxide reduction (Lloyd et al., 2003; Butler et al., 2004; Qian et al., 2007, 2011; Aklujkar et al., 2009; Lovley et al., 2011; Lovley, 2012), current production (Nevin et al., 2009; Inoue et al., 2010), or current uptake on electrodes (Holmes et al., 2006; Strycharz et al., 2011). However, there are slight differences in the types of cytochromes expressed during growth in electron-donating and electron up-taking modes (Strycharz et al., 2011).

*Geobacter sulfurreducens* growing via bDIET with *G. metallireducens* highly expresses an extracellular *c*-type cytochrome, OmcS (Summers et al., 2010; Shrestha et al., 2013a, b). OmcS decorates the pili of *G. sulfurreducens* (Leang et al., 2010; Summers et al., 2010) and is required for bDIET and Fe^3^^+^ reduction (Mehta et al., 2005; Ding et al., 2008; Qian et al., 2011) but not for current production (Nevin et al., 2009). OmcS is not necessary while growing via H_2_ interspecies transfer with *P. carbinolicus* (Rotaru et al., 2012).

Another extracellular cytochrome OmcZ, which helps *G. sulfurreducens* achieve high current densities in single species biofilms (Nevin et al., 2009; Richter et al., 2009), was not required for bDIET in *G. sulfurreducens* – *G. metallireducens* co-cultures (Shrestha et al., 2013b) or during iron oxide reduction (Nevin et al., 2009).

There is no correspondence between the well studied extracellular cytochromes in *G. sulfurreducens* and *G. metallireducens*, and today we have yet no clear understanding, about the exact role of each cytochrome in *G. metallireducens* during extracellular electron transfer processes. And yet it must be noted that extracellular cytochrome like OmcS in the electron acceptor strain, *G. sulfurreducens* were highly relevant for the interspecies association. How exactly they aid the electron transfer process is yet to be uncovered.

#### bDIET in environmental communities

The possible existence of bDIET in the natural ecosystem was first reported by Morita et al. (2011), while studying the mechanism of interspecies electron exchange in the natural methanogenic communities that formed conductive aggregates in a simulated anaerobic wastewater digester converting brewery wastes to methane. The microbial community structure in up-flow anaerobic sludge blanket digester aggregates showed the predominance of *Geobacter* spp. (Morita et al., 2011; Rotaru et al., 2014). It is interesting to note that in most of the methanogenic environments where bDIET is reported, *Geobacter* spp. are abundant (Kato et al., 2012a; Aulenta et al., 2013; Zhou et al., 2013a; Rotaru et al., 2014), which is probably because *Geobacter* spp. form conductive networks using pili (Malvankar et al., 2011; Malvankar and Lovley, 2012) and transfer electrons to methanogens such as *Methanosaeta* (Morita et al., 2011; Rotaru et al., 2014). Similar species abundance has also been reported in enrichment culture converting coal to methane, where *Geobacter* and *Methanosaeta* were the dominant genera (Jones et al., 2010) possibly using coal as an electron donor and an electron transfer mediator.

### MINERAL MEDIATED DIET (mDIET)

The need to produce biological conductive molecular networks can be averted by the addition of conductive minerals (Liu et al., 2012, 2014). mDIET could take place via non-biological conductive networks of semi-conductive minerals (**Figures 2D,E**) like nano-magnetite (Kato et al., 2012a, b; Liu et al., 2014), granulated activated carbon (GAC; Liu et al., 2012) or biochar (Chen et al., 2014) in the absence of molecular conduits.

For example, electrically conductive magnetite nano-particles facilitate mDIET from *G. sulfurreducens* to *Thiobacillus denitrificans*, accomplishing acetate oxidation coupled to nitrate reduction (Kato et al., 2012b). Recently, magnetite nano-particles were shown to compensate for the absence of OmcS on the pili of a deficient *G. sulfurreducens* co-cultured with *G. metallireducens* in the presence of ethanol and fumarate (Liu et al., 2014; **Figure 2D**). Another conductive material, GAC promotes mDIET, bypassing biologically produced electrical conduits (Liu et al., 2012), as evident from the ability to restore syntrophic metabolism in co-cultures deficient in pili or cytochromes (Liu et al., 2012).

#### mDIET in environmental communities

Although extracellular appendages are required for the respiration of extracellular electron acceptors (Reguera et al., 2005; Tremblay et al., 2012), they can be replaced with conductive materials which can mediate electron transfer between cells during mDIET. Naturally occurring minerals could offer ecological advantages because of their abundance in natural ecosystems (Kato et al., 2012b), where they could aid mDIET in the absence of pre-evolved molecular conduits. Iron is one of the most ubiquitous metals in Earth’s crust (Braunschweig et al., 2013) and could act as conductive mediator for mDIET, demanding less energetic investment from the species exchanging electrons because there would be no need to produce extracellular components for biological electrical connections (Kato et al., 2012b). For example, magnetite, a conductive iron (II&III)-oxide, stimulated methane production in rice paddy soils and enriched for *Geobacter* and *Methanosarcina* species, which likely exchanged electrons via magnetite minerals (Kato et al., 2012a; Zhou et al., 2013b). Electrically conductive magnetite (Fe_3_O_4_) nano-particles could also enhance reductive dechlorination of trichloroethane, an ubiquitous groundwater pollutant, by allowing electrons to be transferred extracellular from acetate oxidizing microorganisms to trichloroethane dechlorinating microorganisms (Aulenta et al., 2013). In this study the abundant microorganisms were also *Geobacter* spp., which accounted for 50% of the total bacterial population (Aulenta et al., 2013).

Similarly, it has been reported that poorly crystalline akaganeite (β-polymorph of FeOOH) enhanced mDIET to methanogens in slurries from river sediments (Jiang et al., 2013). In such slurries, *Clostridium* coupled Fe^3^^+^-akaganeite reduction to Fe^2^^+^ with acetate oxidation. Partly, electrons from Fe^2^^+^ were used by the methanogen to convert bicarbonate to methane. Partly, Fe^2^^+^ ions were re-adsorbed onto akaganeite nano-rods, followed by re-precipitation as structural Fe^3^^+^ with the simultaneous formation of goethite (α-polymorph of FeOOH) nanofibres (Jiang et al., 2013).

Anthraquinone disulphonate was also suggested to facilitate mDIET between *Geobacter* spp. and *Methanosarcina* spp. in rice paddies (Zhou et al., 2013b). The impact of AQDS on methanogenesis is in contrast with studies in defined co-cultures of *Geobacter* and *Methanosarcina* (Liu et al., 2012). However, soils are not well-defined systems, and it is possible that in soil other interactions happen between humics and soil components, which should be further investigated.

## IMPLICATIONS

The electron exchange between syntrophic partners growing together by bDIET requires cells to develop efficient conductive biological contacts via pili and cytochromes in the absence of conductive mediators (mDIET). However, little is known about the importance of bDIET/mDIET-based interactions in the environment or in man-made systems. A better understanding could help devise better strategies for wastewater digestion, or to control methane emission in environments where such emission are high, like landfills, or rice paddies.

## Conflict of Interest Statement

The authors declare that the research was conducted in the absence of any commercial or financial relationships that could be construed as a potential conflict of interest.

## References

[B1] Aklujkar M. Krushkal J. Dibartolo G. Lapidus A. Land M. L. Lovley D. R. (2009). The genome sequence of *Geobacter metallireducens*: features of metabolism, physiology and regulation common and dissimilar to *Geobacter sulfurreducens*. *BMC Microbiol.* 9:109 10.1186/1471-2180-9-109 PMC2700814 19473543

[B2] Aulenta F. Rossetti S. Amalfitano S. Majone M. Tandoi V. (2013). Conductive magnetite nanoparticles accelerate the microbial reductive dechlorination of trichloroethene by promoting interspecies electron transfer processes. *ChemSusChem* 6 433–436 10.1002/cssc.201200748 23401476

[B3] Biebl H. Pfennig N. (1978). Growth yields of green sulfur bacteria in mixed cultures with sulfur and sulfate reducing bacteria. *Arch. Microbiol.* 117 9–16 10.1007/Bf00689344

[B4] Bittner M. Hilscherova K. Giesy J. P. (2007). Changes of AhR-mediated activity of humic substances after irradiation. *Environ. Int.* 33 812–816 10.1016/j.envint.2007.03.011 17467800

[B5] Boetius A. Ravenschlag K. Schubert C. J. Rickert D. Widdel F. Gieseke A. (2000). A marine microbial consortium apparently mediating anaerobic oxidation of methane. *Nature* 407 623–626 10.1038/35036572 11034209

[B6] Boone D. R. Johnson R. L. Liu Y. (1989). Diffusion of the interspecies electron carriers H-2 and formate in methanogenic ecosystems and its implications in the measurement of Km for H-2 or formate uptake. *Appl. Environ. Microbiol.* 55 1735–1741 16347966 10.1128/aem.55.7.1735-1741.1989 PMC202943

[B7] Braunschweig J. Bosch J. Meckenstock R. U. (2013). Iron oxide nanoparticles in geomicrobiology: from biogeochemistry to bioremediation. *N. Biotechnol.* 30 793–802 10.1016/j.nbt.2013.03.008 23557995

[B8] Brutinel E. D. Gralnick J. A. (2012). Shuttling happens: soluble flavin mediators of extracellular electron transfer in Shewanella. *Appl. Microbiol. Biotechnol.* 93 41–48 10.1007/s00253-011-3653-0 22072194

[B9] Bryant M. P. Wolin E. A. Wolin M. J. Wolfe R. S. (1967). *Methanobacillus omelianskii*, a symbiotic association of two species of bacteria. *Arch. Mikrobiol.* 59 20–31 10.1007/BF00406313 5602458

[B10] Butler J. E. Kaufmann F. Coppi M. V. Nunez C. Lovley D. R. (2004). MacA, a diheme c-type cytochrome involved in Fe(III) reduction by *Geobacter sulfurreducens*. *J. Bacteriol.* 186 4042–4045 10.1128/JB.186.12.4042-4045.2004 15175321 PMC419948

[B11] Chen S. Rotaru A.-E. Shrestha P. M. Malvankar N. Liu F. Fan W. (2014). Promoting interspecies electron transfer with biochar. *Sci. Rep. (in press)*. 10.1038/srep05019 PMC4028902 24846283

[B12] Conrad R. (2009). The global methane cycle: recent advances in understanding the microbial processes involved. *Environ. Microbiol. Rep.* 1 285–292 10.1111/j.1758-2229.2009.00038.x 23765881

[B13] de Bok F. A. Plugge C. M. Stams A. J. (2004). Interspecies electron transfer in methanogenic propionate degrading consortia. *Water Res.* 38 1368–1375 10.1016/j.watres.2003.11.028 15016514

[B14] Ding Y. H. R. Hixson K. K. Aklujkar M. A. Lipton M. S. Smith R. D. Lovley D. R. (2008). Proteome of *Geobacter sulfurreducens* grown with Fe(III) oxide or Fe(III) citrate as the electron acceptor. *Biochim. Biophys. Acta* 1784 1935–1941 10.1016/j.bbapap.2008.06.011 18638577

[B15] Dong X. Stams A. J. (1995). Evidence for H2 and formate formation during syntrophic butyrate and propionate degradation. *Anaerobe* 1 35–39 10.1016/S1075-9964(95)80405-6 16887505

[B16] Feng Y. Lin X. Yu Y. Zhang H. Chu H. Zhu J. (2013). Elevated ground-level O_3_ negatively influences paddy methanogenic archaeal community. *Sci. Rep.* 3 3193 10.1038/srep03193 PMC3824163 24217205

[B17] Grosskopf R. Janssen P. H. Liesack W. (1998). Diversity and structure of the methanogenic community in anoxic rice paddy soil microcosms as examined by cultivation and direct 16S rRNA gene sequence retrieval. *Appl. Environ. Microbiol.* 64 960–969 9501436 10.1128/aem.64.3.960-969.1998 PMC106352

[B18] Hattori S. Luo H. Shoun H. Kamagata Y. (2001). Involvement of formate as an interspecies electron carrier in a syntrophic acetate-oxidizing anaerobic microorganism in coculture with methanogens. *J. Biosci. Bioeng.* 91 294–298 16232992 10.1263/jbb.91.294

[B19] Holmes D. E. Chaudhuri S. K. Nevin K. P. Mehta T. Methe B. A. Liu A. (2006). Microarray and genetic analysis of electron transfer to electrodes in *Geobacter sulfurreducens*. *Environ. Microbiol.* 8 1805–1815 10.1111/j.1462-2920.2006.01065.x 16958761

[B20] Inoue K. Qian X. Morgado L. Kim B. C. Mester T. Izallalen M. (2010). Purification and characterization of OmcZ, an outer-surface, octaheme c-type cytochrome essential for optimal current production by *Geobacter sulfurreducens*. *Appl. Environ. Microbiol.* 76 3999–4007 10.1128/AEM.00027-10 20400562 PMC2893489

[B21] Jiang S. Park S. Yoon Y. Lee J. H. Wu W. M. Phuoc Dan N. (2013). Methanogenesis facilitated by geobiochemical iron cycle in a novel syntrophic methanogenic microbial community. *Environ. Sci. Technol.* 47 10078–10084 10.1021/es402412c 23919295

[B22] Jones E. J. P. Voytek M. A. Corum M. D. Orem W. H. (2010). Stimulation of methane generation from nonproductive coal by addition of nutrients or a microbial consortium. *Appl. Environ. Microbiol.* 76 7013–7022 10.1128/Aem.00728-10 20817801 PMC2976240

[B23] Kaden J. Galushko A. S. Schink B. (2002). Cysteine-mediated electron transfer in syntrophic acetate oxidation by cocultures of *Geobacter sulfurreducens* and *Wolinella succinogenes*. *Arch. Microbiol.* 178 53–58 10.1007/s00203-002-0425-3 12070769

[B24] Kato S. Hashimoto K. Watanabe K. (2012a). Methanogenesis facilitated by electric syntrophy via (semi)conductive iron-oxide minerals. *Environ. Microbiol.* 14 1646–1654 10.1111/j.1462-2920.2011.02611.x 22004041

[B25] Kato S. Hashimoto K. Watanabe K. (2012b). Microbial interspecies electron transfer via electric currents through conductive minerals. *Proc. Natl. Acad. Sci. U.S.A.* 109 10042–10046 10.1073/pnas.1117592109 22665802 PMC3382511

[B26] Kleerebezem R. Hulshoff Pol L. W. Lettinga G. (1999). Anaerobic degradation of phthalate isomers by methanogenic consortia. *Appl. Environ. Microbiol.* 65 1152–1160 10049876 10.1128/aem.65.3.1152-1160.1999 PMC91157

[B27] Leang C. Qian X. L. Mester T. Lovley D. R. (2010). Alignment of the c-type cytochrome OmcS along pili of *Geobacter sulfurreducens*. *Appl. Environ. Microbiol.* 76 4080–4084 10.1128/Aem.00023-10 20400557 PMC2893476

[B28] Liu F. H. Rotaru A.-E. Shrestha P. M. Malvankar N. S. Nevin K. P. Lovley D. R. (2012). Promoting direct interspecies electron transfer with activated carbon. *Energy Environ. Sci.* 5 8982–8989 10.1039/C2ee22459c

[B29] Liu F. Rotaru A.-E. Shrestha P. M. Malvankar N. S. Nevin K. Lovley D. (2014). Magnetite compensates for the lack of a pilin-associated c-type cytochrome in extracellular electron exchange. *Environ. Microbiol.* 10.1111/1462-2920.12485 [Epub ahead of print]. 24725505

[B30] Liu X. Tremblay P. L. Malvankar N. S. Nevin K. P. Lovley D. R. Vargas M. (2013). *Geobacter sulfurreducens* strain expressing *Pseudomonas aeruginosa* type IV pili localizes OmcS on pili but is deficient in Fe(III) oxide reduction and current production. *Appl. Environ. Microbiol.* 80 1219–1224 10.1128/AEM.0293813 24296506 PMC3911229

[B31] Lloyd J. R. Leang C. Hodges Myerson A. L. Coppi M. V. Cuifo S. Methe B. (2003). Biochemical and genetic characterization of PpcA, a periplasmic c-type cytochrome in *Geobacter sulfurreducens*. *Biochem. J.* 369 153–161 10.1042/BJ20020597 12356333 PMC1223068

[B32] Lovley D. R. (2011). Live wires: direct extracellular electron exchange for bioenergy and the bioremediation of energy-related contamination. *Energy Environ. Sci.* 4 4896–4906 10.1039/C1ee02229f

[B33] Lovley D. R. (2012). Long-range electron transport to Fe(III) oxide via pili with metallic-like conductivity. *Biochem. Soc. Trans.* 40 1186–1190 10.1042/BST20120131 23176452

[B34] Lovley D. R. Coates J. D. Bluntharris E. L. Phillips E. J. P. Woodward J. C. (1996). Humic substances as electron acceptors for microbial respiration. *Nature* 382 445–448 10.1038/382445a0

[B35] Lovley D. R. Ferry J. G. (1985). Production and consumption of H2 during growth of *Methanosarcina* spp. on acetate. *Appl. Environ. Microbiol.* 49 247–249 16346703 10.1128/aem.49.1.247-249.1985 PMC238382

[B36] Lovley D. R. Fraga J. L. Blunt-Harris E. L. Hayes L. A. Phillips E. J. P. Coates J. D. (1998). Humic substances as a mediator for microbially catalyzed metal reduction. *Acta Hydrochim. Hydrobiol.* 26 152–157 10.1002/(Sici)1521-401x(199805)26:3<152::Aid-Aheh152>3.0.Co;2-D

[B37] Lovley D. R. Fraga J. L. Coates J. D. Blunt-Harris E. L. (1999). Humics as an electron donor for anaerobic respiration. *Environ. Microbiol.* 1 89–98 10.1046/j.1462-2920.1999.00009.x 11207721

[B38] Lovley D. R. Ueki T. Zhang T. Malvankar N. S. Shrestha P. M. Flanagan K. A. (2011). *Geobacter*: the microbe electric’s physiology, ecology, and practical applications. *Adv. Microb. Physiol.* 59 1–100 10.1016/B978-0-12-387661-4.00004-5 22114840

[B39] Malvankar N. S. Lovley D. R. (2012). Microbial nanowires: a new paradigm for biological electron transfer and bioelectronics. *ChemSusChem* 5 1039–1046 10.1002/cssc.201100733 22614997

[B40] Malvankar N. S. Lovley D. R. (2014). Microbial nanowires for bioenergy applications. *Curr. Opin. Biotechnol.* 27 88–95 10.1016/j.copbio.2013.12.003 24863901

[B41] Malvankar N. S. Vargas M. Nevin K. P. Franks A. E. Leang C. Kim B. C. (2011). Tunable metallic-like conductivity in microbial nanowire networks. *Nat. Nanotechnol.* 6 573–579 10.1038/Nnano.2011.119 21822253

[B42] Marsili E. Baron D. B. Shikhare I. D. Coursolle D. Gralnick J. A. Bond D. R. (2008). Shewanella secretes flavins that mediate extracellular electron transfer. *Proc. Natl. Acad. Sci. U.S.A.* 105 3968–3973 10.1073/pnas.0710525105 18316736 PMC2268775

[B43] McInerney M. J. Sieber J. R. Gunsalus R. P. (2011). Microbial syntrophy: ecosystem-level biochemical cooperation. *Microbe* 6 479–485

[B44] Mehta T. Coppi M. V. Childers S. E. Lovley D. R. (2005). Outer membrane c-type cytochromes required for Fe(III) and Mn(IV) oxide reduction in *Geobacter sulfurreducens*. *Appl. Environ. Microbiol.* 71 8634–8641 10.1128/AEM.71.12.8634-8641.2005 16332857 PMC1317342

[B45] Milucka J. Ferdelman T. G. Polerecky L. Franzke D. Wegener G. Schmid M. (2012). Zero-valent sulphur is a key intermediate in marine methane oxidation. *Nature* 491 541–546 10.1038/Nature11656 23135396

[B46] Moran J. J. Beal E. J. Vrentas J. M. Orphan V. J. Freeman K. H. House C. H. (2008). Methyl sulfides as intermediates in the anaerobic oxidation of methane. *Environ. Microbiol.* 10 162–173 10.1111/j.1462-2920.2007.01441.x 17903217

[B47] Moreira C. G. Palmer K. Whiteley M. Sircili M. P. Trabulsi L. R. Castro A. F. P. (2006). Bundle-forming pili and EspA are involved in biofilm formation by enteropathogenic *Escherichia coli*. *J. Bacteriol.* 188 3952–3961 10.1128/Jb.00177-06 16707687 PMC1482920

[B48] Morita M. Malvankar N. S. Franks A. E. Summers Z. M. Giloteaux L. Rotaru A.-E. (2011). Potential for direct interspecies electron transfer in methanogenic wastewater digester aggregates. *MBio* 2 e00159–e00111 10.1128/mBio.00159-11 21862629 PMC3157894

[B49] Morris B. E. Henneberger R. Huber H. Moissl-Eichinger C. (2013). Microbial syntrophy: interaction for the common good. *FEMS Microbiol. Rev.* 37 384–406 10.1111/1574-6976.12019 23480449

[B50] Nagarajan H. Embree M. Rotaru A.-E. Shrestha P. M. Feist A. M. Palsson B. O. (2013). Characterization and modelling of interspecies electron transfer mechanisms and microbial community dynamics of a syntrophic association. *Nat. Commun.* 4 2809 10.1038/ncomms3809 24264237

[B51] Nedwell D. B. Banat I. M. (1981). Hydrogen as an electron-donor for sulfate-reducing bacteria in slurries of salt-marsh sediment. *Microb. Ecol.* 7 305–313 10.1007/Bf02341425 24227546

[B52] Nevin K. P. Kim B. C. Glaven R. H. Johnson J. P. Woodard T. L. Methe B. A. (2009). Anode biofilm transcriptomics reveals outer surface components essential for high density current production in *Geobacter sulfurreducens* fuel cells. *PLoS ONE* 4:e5628 10.1371/journal.pone.0005628 PMC2680965 19461962

[B53] Newman D. K. Kolter R. (2000). A role for excreted quinones in extracellular electron transfer. *Nature* 405 94–97 10.1038/35011098 10811225

[B54] Oxaran V. Ledue-Clier F. Dieye Y. Herry J. M. Pechoux C. Meylheuc T. (2012). Pilus biogenesis in *Lactococcus lactis*: molecular characterization and role in aggregation and biofilm formation. *PLoS ONE* 7:e50989 10.1371/journal.pone.0050989 PMC3516528 23236417

[B55] Qian X. L. Mester T. Morgado L. Arakawa T. Sharma M. L. Inoue K. (2011). Biochemical characterization of purified OmcS, a c-type cytochrome required for insoluble Fe(III) reduction in *Geobacter sulfurreducens*. *Biochim. Biophys. Acta* 1807 404–412 10.1016/j.bbabio.2011.01.003 21236241

[B56] Qian X. L. Reguera G. Mester T. Lovley D. R. (2007). Evidence that OmcB and OmpB of *Geobacter sulfurreducens* are outer membrane surface proteins. *FEMS Microbiol. Lett.* 277 21–27 10.1111/j.1574-6968.2007.00915.x 17986080

[B57] Reguera G. Mccarthy K. D. Mehta T. Nicoll J. S. Tuominen M. T. Lovley D. R. (2005). Extracellular electron transfer via microbial nanowires. *Nature* 435 1098–1101 10.1038/Nature03661 15973408

[B58] Reguera G. Nevin K. P. Nicoll J. S. Covalla S. F. Woodard T. L. Lovley D. R. (2006). Biofilm and nanowire production leads to increased current in *Geobacter sulfurreducens* fuel cells. *Appl. Environ. Microbiol.* 72 7345–7348 10.1128/AEM.01444-06 16936064 PMC1636155

[B59] Reguera G. Pollina R. B. Nicoll J. S. Lovley D. R. (2007). Possible nonconductive role of *Geobacter sulfurreducens* pilus nanowires in biofilm formation. *J. Bacteriol.* 189 2125–2127 10.1128/Jb.01284-06 17158673 PMC1855775

[B60] Richter H. Nevin K. P. Jia H. Lowy D. A. Lovley D. R. Tender L. M. (2009). Cyclic voltammetry of biofilms of wild type and mutant *Geobacter sulfurreducens* on fuel cell anodes indicates possible roles of OmcB, OmcZ, type IV pili, and protons in extracellular electron transfer. *Energy Environ. Sci.* 2 506–516 10.1039/b816647a

[B61] Rotaru A.-E. Shrestha P. M. Liu F. Shrestha M. Shrestha D. Embree M. (2014). A new model for electron flow during anaerobic digestion: direct interspecies electron transfer to Methanosaeta for the reduction of carbon dioxide to methane. *Energy Environ. Sci.* 7 408–415 10.1039/c3ee42189a

[B62] Rotaru A.-E. Shrestha P. M. Liu F. Ueki T. Nevin K. Summers Z. M. (2012). Interspecies electron transfer via hydrogen and formate rather than direct electrical connections in cocultures of *Pelobacter carbinolicus* and *Geobacter sulfurreducens*. *Appl. Environ. Microbiol.* 78 7645–7651 10.1128/AEM.01946-12 22923399 PMC3485699

[B63] Schink B Stams A. J. M. (2006). *Syntrophism among Prokaryotes. Prokaryotes: A Handbook on the Biology of Bacteria* 3rd Edn Vol. 2 (New York: Springer Verlag) 309–335

[B64] Schink B. Stams A. M. (2013). “Syntrophism Among Prokaryotes,” in *The Prokaryotes* eds Rosenberg E. Delong E. Lory S. Stackebrandt E. Thompson F. (New York: Springer Berlin Heidelberg) 471–493

[B65] Schut G. J Adams M. W. W. (2009). The iron-hydrogenase of thermotoga maritima utilizes ferredoxin and NADH synergistically: a new perspective on anaerobic hydrogen production. *J. Bacteriol.* 191 4451–4457 10.1128/Jb.01582-08 19411328 PMC2698477

[B66] Shrestha P. M. Rotaru A.-E. Aklujkar M. Liu F. Shrestha M. Summers Z. M. (2013a). Syntrophic growth with direct interspecies electron transfer as the primary mechanism for energy exchange. *Environ. Microbiol. Rep.* 5 904–910 10.1111/1758-2229.12093 24249299

[B67] Shrestha P. M. Rotaru A.-E. Summers Z. M. Shrestha M. Liu F. Lovley D. R. (2013b). Transcriptomic and genetic analysis of direct interspecies electron transfer. *Appl. Environ. Microbiol.* 79 2397–2404 10.1128/AEM.03837-12 23377933 PMC3623256

[B68] Sieber J. R. Le H. M. Mcinerney M. J. (2014). The importance of hydrogen and formate transfer for syntrophic fatty, aromatic and alicyclic metabolism. *Environ. Microbiol.* 16 177–188 10.1111/1462-2920.12269 24387041

[B69] Sieber J. R. Mcinerney M. J. Gunsalus R. P. (2012). Genomic insights into syntrophy: the paradigm for anaerobic metabolic cooperation. *Annu. Rev. Microbiol.* 66 429–452 10.1146/annurev-micro-090110-102844 22803797

[B70] Sieber J. R. Sims D. R. Han C. Kim E. Lykidis A. Lapidus A. L. (2010). The genome of *Syntrophomonas wolfei*: new insights into syntrophic metabolism and biohydrogen production. *Environ. Microbiol.* 12 2289–2301 10.1111/j.1462-2920.2010.02237.x 21966920

[B71] Snider R. M. Strycharz-Glaven S. M. Tsoi S. D. Erickson J. S. Tender L. M. (2012). Long-range electron transport in *Geobacter sulfurreducens* biofilms is redox gradient-driven. *Proc. Natl. Acad. Sci. U.S.A.* 109 15467–15472 10.1073/pnas.1209829109 22955881 PMC3458377

[B72] Sousa D. Z. Smidt H. Alves M. M. Stams A. J. M. (2007). *Syntrophomonas zehnderi* sp nov., an anaerobe that degrades long-chain fatty acids in co-culture with *Methanobacterium formicicum*. *Int. J. Syst. Evol. Microbiol*. 57 609–615 10.1099/ijs.0.647340 17329794

[B73] Stams A. J. De Bok F. A. Plugge C. M. Van Eekert M. H. Dolfing J. Schraa G. (2006). Exocellular electron transfer in anaerobic microbial communities. *Environ. Microbiol.* 8 371–382 10.1111/j.1462-2920.2006.00989.x 16478444

[B74] Stams A. J. Plugge C. M. (2009). Electron transfer in syntrophic communities of anaerobic bacteria and archaea. *Nat. Rev. Microbiol.* 7 568–577 10.1038/nrmicro2166 19609258

[B75] Strycharz-Glaven S. M. Snider R. M. Guiseppi-Elie A. Tender L. M. (2011). On the electrical conductivity of microbial nanowires and biofilms. *Energy Environ. Sci.* 4 4366–4379 10.1039/c1ee01753e

[B76] Strycharz S. M. Glaven R. H. Coppi M. V. Gannon S. M. Perpetua L. A. Liu A. (2011). Gene expression and deletion analysis of mechanisms for electron transfer from electrodes to *Geobacter sulfurreducens*. *Bioelectrochemistry* 80 142–150 10.1016/j.bioelechem.2010.07.005 20696622

[B77] Summers Z. M. Fogarty H. E. Leang C. Franks A. E. Malvankar N. S. Lovley D. R. (2010). Direct exchange of electrons within aggregates of an evolved syntrophic coculture of anaerobic bacteria. *Science* 330 1413–1415 10.1126/science.1196526 21127257

[B78] Thiele J. H. Zeikus J. G. (1988). Control of interspecies electron flow during anaerobic digestion: significance of formate transfer versus hydrogen transfer during syntrophic methanogenesis in flocs. *Appl. Environ. Microbiol.* 54 20–29 16347526 10.1128/aem.54.1.20-29.1988 PMC202391

[B79] Tremblay P. L. Aklujkar M. Leang C. Nevin K. P. Lovley D. (2012). A genetic system for *Geobacter metallireducens*: role of the flagellin and pilin in the reduction of Fe(III) oxide. *Environ. Microbiol. Rep.* 4 82–88 10.1111/j.1758-2229.2011.00305.x 23757233

[B80] Ueki T. Lovley D. R. (2010). Genome-wide gene regulation of biosynthesis and energy generation by a novel transcriptional repressor in *Geobacter* species. *Nucleic Acids Res.* 38 810–821 10.1093/nar/gkp1085 19939938 PMC2817479

[B81] Vargas M. Malvankar N. S. Tremblay P. L. Leang C. Smith J. A. Patel P. (2013). Aromatic amino acids required for pili conductivity and long-range extracellular electron transport in *Geobacter sulfurreducens*. *MBio* 4 e00105–e00113 10.1128/mBio.00105-13 23481602 PMC3604773

[B82] Vavilin V. A. Qu X. Mazeas L. Lemunier M. Duquennoi C. He P. J. (2008). Methanosarcina as the dominant aceticlastic methanogens during mesophilic anaerobic digestion of putrescible waste. *Antonie Van Leeuwenhoek* 94 593–605 10.1007/s10482-008-9279-2 18791805

[B83] von Canstein H. Ogawa J. Shimizu S. Lloyd J. R. (2008). Secretion of flavins by Shewanella species and their role in extracellular electron transfer. *Appl. Environ. Microbiol.* 74 615–623 10.1128/Aem.01387-07 18065612 PMC2227709

[B84] Wintermute E. H. Silver P. A. (2010). Dynamics in the mixed microbial concourse. *Genes Dev.* 24 2603–2614 10.1101/gad.1985210 21123647 PMC2994034

[B85] Ying Y. Yu K. Xia Y. Lau F. T. Tang D. T. Fung W. C. (2014). Metagenomic analysis of sludge from full-scale anaerobic digesters operated in municipal wastewater treatment plants. *Appl. Microbiol. Biotechnol.*. 10.1007/s00253-014-5648-0 [Epub ahead of print]. 24633414

[B86] Zhou M. Chen J. Freguia S. Rabaey K. Keller J. (2013a). Carbon and electron fluxes during the electricity driven 1,3-propanediol biosynthesis from glycerol. *Environ. Sci. Technol.* 47 11199–11205 10.1021/es402132r 23947779

[B87] Zhou S. Xu J. Yang G. Zhuang L. (2013b). Methanogenesis affected by the co-occurrence of iron(III) oxides and humic substances. *FEMS Microbiol. Ecol.* 88 107–120 10.1111/1574-6941.12274 24372096

[B88] Zindel U. Freudenberg W. Rieth M. Andreesen J. R. Schnell J. Widdel F. (1988). Eubacterium-acidaminophilum Sp-nov, a versatile amino acid-degrading anaerobe producing or utilizing H-2 or formate – description and enzymatic studies. *Arch. Microbiol.* 150 254–266 10.1007/Bf00407789

